# Bis[2-({[2-(methyl­sulfan­yl)phen­yl]imino}­meth­yl)phenolato-κ^2^ *N*,*O*]zinc chloro­form disolvate

**DOI:** 10.1107/S1600536812045473

**Published:** 2012-11-17

**Authors:** Yen-Jen Chen, Mon-Wei Hsiao, Nai-Yuan Jheng, Yi-Chun Lai, Hsuan-Ying Chen

**Affiliations:** a Department of Medicinal and Applied Chemistry, Kaohsiung Medical University, Kaohsiung 807, Taiwan; b Department of Chemistry, National Chung Hsing University, Taichung 402, Taiwan

## Abstract

The monomeric title complex, [Zn(C_14_H_12_NOS)_2_]·2CHCl_3_ or *L*_2_Zn·2CHCl_3_, where *L* is the 2-({[2-(methyl­sulfan­yl)phen­yl]imino}­meth­yl)phenolate anion, may be obtained by the reaction of *L*ZnEt with benzyl alcohol or by the reaction of two equivalents of *L*H with ZnEt_2_ in tetra­hydro­furan. The Zn atom, located on a twofold axis, is four-coordinated in a distorted tetra­hedral geometry by two O atoms [Zn—O = 1.9472 (19) Å] from the phenolate anions and two imine N atoms [Zn—N = 2.054 (2) Å].

## Related literature

For backgroud to poly(lactide) (PLA) and its copolymers, see: Huang *et al.* (2007 ▶). For the use of bulky ligands coordinated to the active metal centre to avoid undesirable transesterification during synthesis by ring-opening polymerization (ROP) of lactides, see: Wu *et al.* (2006 ▶). Many complexes with bulky ligands have been designed for this function, incorporating a single active metal site, see: Wu *et al.* (2006 ▶). For the preparation of a series of Zn complexes with *N*,*N*,*O*-tridentate Schiff bases, which have great activity in the ROP of lactides, see: Chen *et al.* (2006 ▶). For the 2-(2,6-diisopropyl­phenyl­imino)­meth­yl)-4-nitro­phenolate anion, see: Chisholm *et al.* (2001 ▶). 
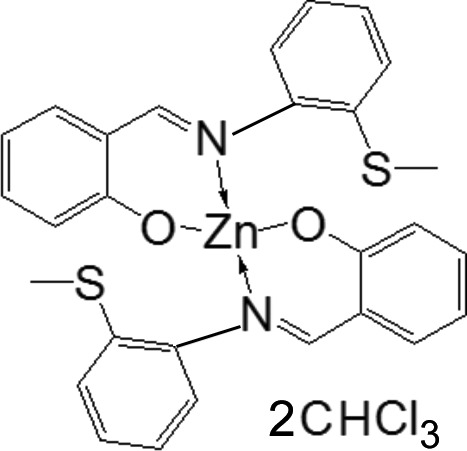


## Experimental

### 

#### Crystal data

[Zn(C_14_H_12_NOS)_2_]·2CHCl_3_
*M* *_r_* = 788.72
Monoclinic, 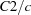

*a* = 10.5673 (9) Å
*b* = 21.5085 (19) Å
*c* = 15.1215 (14) Å
β = 97.309 (2)°
*V* = 3409.0 (5) Å^3^
*Z* = 4
Mo *K*α radiation
μ = 1.34 mm^−1^
*T* = 110 K
0.45 × 0.38 × 0.32 mm

#### Data collection

Bruker SMART CCD area-detector diffractometer
Absorption correction: multi-scan (*SADABS*; Bruker, 2001 ▶) *T*_min_ = 0.381, *T*_max_ = 1.000
9547 measured reflections
3345 independent reflections
2494 reflections with *I* > 2σ(*I*)
*R*_int_ = 0.037

#### Refinement

*R*[*F* ^2^ > 2σ(*F* ^2^)] = 0.044
*wR*(*F* ^2^) = 0.135
*S* = 1.01
3345 reflections
195 parameters
6 restraints
H-atom parameters constrained
Δρ_max_ = 0.50 e Å^−3^
Δρ_min_ = −0.50 e Å^−3^

### 

Data collection: *APEX2* (Bruker, 2007 ▶); cell refinement: *SAINT* (Bruker, 2007 ▶); data reduction: *SAINT*; program(s) used to solve structure: *SHELXS97* (Sheldrick, 2008 ▶); program(s) used to refine structure: *SHELXL97* (Sheldrick, 2008 ▶); molecular graphics: *SHELXTL* (Sheldrick, 2008 ▶); software used to prepare material for publication: *SHELXTL*.

## Supplementary Material

Crystal structure: contains datablock(s) I, global. DOI: 10.1107/S1600536812045473/hp2049sup1.cif

Structure factors: contains datablock(s) I. DOI: 10.1107/S1600536812045473/hp2049Isup2.hkl

Additional supplementary materials:  crystallographic information; 3D view; checkCIF report

## Figures and Tables

**Fig. 1.**
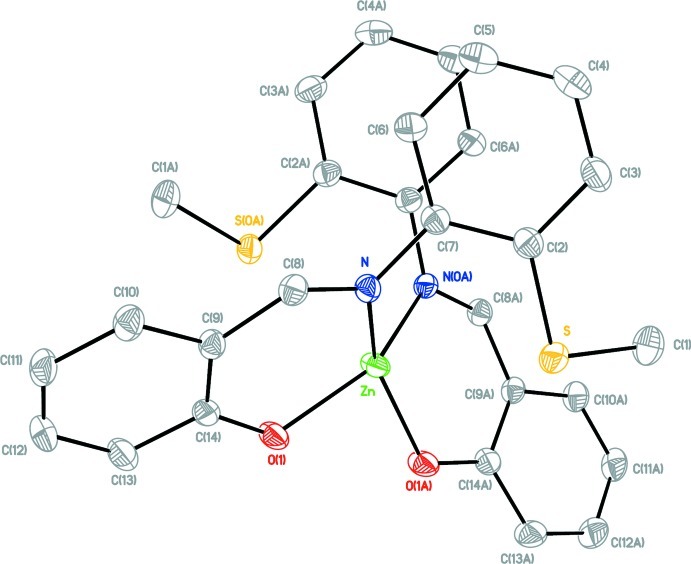
A view of the molecular structure of [L2Zn] with displacement ellipsoids shown at the 20% probability level.

**Fig. 2.**
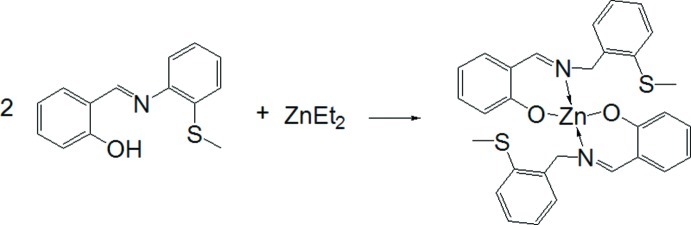
Reaction scheme.

## supplementary crystallographic information

### Comment

Because of their potential applications in many fields, poly(lactide) (PLA) and its copolymers have been investigated intensively (Huang *et al.*, 2007). Ring-opening polymerization (ROP) of lactides is the major method used to synthesize these polymers. In these processes, undesirable transesterification reaction is the drawback but it can be lessened by using bulky ligands coordinated to the active metal centre (Wu *et al.*, 2006). A lot of complexes with bulky ligands have been designed for this function, incorporating a single active metal site (Wu *et al.*, 2006). Lin group have prepared a series of Zn complexes with NNO-tridentate Schiff base supported (Chen *et al.*, 2006) which have great activity in the ROP of lactides. Recently, we have prepared NOS– tridentate Schiff base ligand (2-(((2-methylthiophenyl)methylimino)methyl)phenol) and its Zn complex. During these studies, it has been observed that LZnEt, where *L* is the (2-(((2-methylthiophenyl)methylimino)methyl)phenolate anion (C_14_H_12_NOS), reacts with benzyl alcohol to give *L*_2_Zn, (I) because of disproportionation. It seems that the sulfur atom can not stabilize Zn atom to form Zn alkoxide complex. *L*_2_Zn can also be prepared by the reaction of 2 equal LH with ZnEt_2_ in tetrahydrofuran. In the solid state, complex (I) shows a monomeric structure in which Zn atom are tetracoordinated and the geometry around Zn, resemble distorted tetrahedral with N—Zn—O(1), O(1)—Zn—O(1 A), and N—Zn—N(0 A) bond angles of 93.13 (9)*^o^*, 88.57 (11)*^o^*, and 103.73 (12)*^o^*. The distances of Zn—O and Zn—N are 1.9472 (18) and 2.054 (2) Å. A closely comparable conformation has been observed for the *L*'_2_Zn, where *L*' are the 2-(2,6-diisopropylphenylimino)methyl)-4-nitrophenolate anion (Chisholm *et al.*, 2001) and 2-(2-dimethylaminoethylimino)methyl)-4-bromophenolate anion (Chen *et al.*, 2006).

### Experimental

To a suspension of LH (4.86 g, 20 mmol) in tetrahydrofuran (15 ml) was added ZnEt_2_ (1.22 g, 10 mmol). After being stirred for 3 hr, volatile materials were then removed under a vacuum to yield a yellow powder. The powder was washed twice with hexane (30 ml), and a high yellow powder was obtained after filtration. The crystal was obtain in CHCl_3_ soultion. A colourless crystal was selected from this sample.

### Refinement

X-ray experimental: Data were collected at 173 K on a Siemens *SMART* PLATFORM equipped with A CCD area detector and a graphite monochromator utilizing MoKaradiation (l= 0.71073 Å).Cell parameters were refined using up to 8192 reflections. A full sphere of data (1850 frames) was collected using the w-scan method (0.3°frame width).The first 50 frames were re-measured at the end of data collection to monitor instrument and crystal stability (maximum correction on I was < 1%).Absorption corrections by integration were applied based on measured indexed crystal faces.

The structure was solved by the Direct Methods in *SHELXTL6*, and refined using full-matrix least squares.The non-H atoms were treated anisotropically, whereas the hydrogen atoms were calculated in ideal positions and were riding on their respective carbon atoms.A total of 195 parameters were refined in the final cycle of refinement using 3345 reflections with I > 2 s(I) to yield *R*_1_ and w*R*_2_ of 4.37% and 12.30%, respectively.Refinement was done using F^2^.

### Crystal data

**Table:**

[Zn(C_14_H_12_NOS)_2_]·2CHCl_3_	*F*(000) = 1600
*M**_r_* = 788.72	*D*_x_ = 1.537 Mg m^−^^3^
Monoclinic, *C*2/*c*	Mo *K*α radiation, λ = 0.71073 Å
Hall symbol: -C 2yc	Cell parameters from 4002 reflections
*a* = 10.5673 (9) Å	θ = 2.4–25.8°
*b* = 21.5085 (19) Å	µ = 1.34 mm^−^^1^
*c* = 15.1215 (14) Å	*T* = 110 K
β = 97.309 (2)°	Parallelpiped, yellow
*V* = 3409.0 (5) Å^3^	0.45 × 0.38 × 0.32 mm
*Z* = 4	

### Data collection

**Table:**

Bruker SMART CCD area-detector diffractometer	3345 independent reflections
Radiation source: fine-focus sealed tube	2494 reflections with *I* > 2σ(*I*)
Graphite monochromator	*R*_int_ = 0.037
Detector resolution: 16.0690 pixels mm^-1^	θ_max_ = 26.0°, θ_min_ = 2.2°
phi and ω scans	*h* = −12→13
Absorption correction: multi-scan (*SADABS*; Bruker, 2001)	*k* = −26→23
*T*_min_ = 0.381, *T*_max_ = 1.000	*l* = −18→10
9547 measured reflections	

### Refinement

**Table:**

Refinement on *F*^2^	Primary atom site location: structure-invariant direct methods
Least-squares matrix: full	Secondary atom site location: difference Fourier map
*R*[*F*^2^ > 2σ(*F*^2^)] = 0.044	Hydrogen site location: inferred from neighbouring sites
*wR*(*F*^2^) = 0.135	H-atom parameters constrained
*S* = 1.01	*w* = 1/[σ^2^(*F*_o_^2^) + (0.090*P*)^2^] where *P* = (*F*_o_^2^ + 2*F*_c_^2^)/3
3345 reflections	(Δ/σ)_max_ < 0.001
195 parameters	Δρ_max_ = 0.50 e Å^−^^3^
6 restraints	Δρ_min_ = −0.50 e Å^−^^3^

### Special details

**Table:**

Geometry. All e.s.d.'s (except the e.s.d. in the dihedral angle between two l.s. planes) are estimated using the full covariance matrix. The cell e.s.d.'s are taken into account individually in the estimation of e.s.d.'s in distances, angles and torsion angles; correlations between e.s.d.'s in cell parameters are only used when they are defined by crystal symmetry. An approximate (isotropic) treatment of cell e.s.d.'s is used for estimating e.s.d.'s involving l.s. planes.
Refinement. Refinement of *F*^2^ against ALL reflections. The weighted *R*-factor *wR* and goodness of fit *S* are based on *F*^2^, conventional *R*-factors *R* are based on *F*, with *F* set to zero for negative *F*^2^. The threshold expression of *F*^2^ > σ(*F*^2^) is used only for calculating *R*-factors(gt) *etc*. and is not relevant to the choice of reflections for refinement. *R*-factors based on *F*^2^ are statistically about twice as large as those based on *F*, and *R*- factors based on ALL data will be even larger.

### Fractional atomic coordinates and isotropic or equivalent isotropic displacement parameters (Å^2^)

**Table:**

	*x*	*y*	*z*	*U*_iso_*/*U*_eq_	
Zn	0.0000	0.23879 (2)	0.2500	0.0481 (2)	
S	0.19745 (7)	0.19012 (4)	0.13833 (6)	0.0531 (2)	
N	−0.0763 (2)	0.17983 (10)	0.15038 (15)	0.0398 (5)	
O	−0.12511 (19)	0.30361 (9)	0.21523 (16)	0.0571 (6)	
C1	0.3508 (3)	0.16784 (19)	0.1930 (3)	0.0707 (10)	
H1A	0.4055	0.2036	0.1999	0.106*	
H1B	0.3874	0.1371	0.1577	0.106*	
H1C	0.3419	0.1508	0.2505	0.106*	
C2	0.1132 (3)	0.11931 (14)	0.1323 (2)	0.0482 (7)	
C3	0.1706 (3)	0.06183 (16)	0.1214 (2)	0.0610 (9)	
H3A	0.2578	0.0599	0.1180	0.073*	
C4	0.0998 (4)	0.00797 (16)	0.1157 (3)	0.0752 (11)	
H4A	0.1393	−0.0300	0.1084	0.090*	
C5	−0.0301 (4)	0.01012 (16)	0.1208 (3)	0.0751 (11)	
H5A	−0.0777	−0.0264	0.1174	0.090*	
C6	−0.0886 (3)	0.06656 (14)	0.1308 (2)	0.0557 (8)	
H6A	−0.1758	0.0680	0.1343	0.067*	
C7	−0.0181 (3)	0.12148 (13)	0.13598 (19)	0.0441 (6)	
C8	−0.1873 (3)	0.19109 (14)	0.10609 (19)	0.0447 (7)	
H8A	−0.2161	0.1624	0.0621	0.054*	
C9	−0.2703 (3)	0.24188 (13)	0.11654 (19)	0.0418 (6)	
C10	−0.3943 (3)	0.23780 (16)	0.0687 (2)	0.0537 (8)	
H10A	−0.4142	0.2039	0.0313	0.064*	
C11	−0.4851 (3)	0.28157 (17)	0.0753 (2)	0.0608 (9)	
H11A	−0.5661	0.2776	0.0436	0.073*	
C12	−0.4544 (3)	0.33205 (18)	0.1302 (3)	0.0703 (10)	
H12A	−0.5162	0.3621	0.1358	0.084*	
C13	−0.3341 (3)	0.33914 (15)	0.1770 (3)	0.0638 (9)	
H13A	−0.3163	0.3741	0.2127	0.077*	
C14	−0.2387 (3)	0.29454 (13)	0.1715 (2)	0.0463 (7)	
Cl3	−0.58113 (11)	−0.07026 (6)	0.05933 (10)	0.1060 (4)	
Cl2	−0.34051 (11)	−0.13281 (7)	0.06052 (9)	0.0998 (4)	
Cl1	−0.36432 (13)	−0.03130 (6)	0.18120 (10)	0.1029 (4)	
C30	−0.4421 (3)	−0.09453 (17)	0.1255 (3)	0.0655 (9)	
H30A	−0.4660	−0.1238	0.1702	0.079*	

### Atomic displacement parameters (Å^2^)

**Table:**

	*U*^11^	*U*^22^	*U*^33^	*U*^12^	*U*^13^	*U*^23^
Zn	0.0422 (3)	0.0320 (3)	0.0641 (4)	0.000	−0.0162 (2)	0.000
S	0.0397 (4)	0.0542 (5)	0.0646 (5)	−0.0025 (3)	0.0033 (3)	0.0053 (4)
N	0.0338 (10)	0.0403 (12)	0.0442 (13)	0.0013 (9)	0.0003 (9)	−0.0022 (10)
O	0.0470 (11)	0.0355 (10)	0.0823 (16)	0.0041 (9)	−0.0174 (11)	0.0007 (10)
C1	0.0438 (17)	0.089 (3)	0.077 (2)	0.0010 (17)	−0.0032 (17)	−0.001 (2)
C2	0.0471 (15)	0.0475 (16)	0.0490 (17)	0.0050 (13)	0.0027 (13)	0.0027 (14)
C3	0.0593 (18)	0.0548 (19)	0.070 (2)	0.0158 (15)	0.0132 (17)	−0.0005 (17)
C4	0.087 (3)	0.0436 (18)	0.096 (3)	0.0141 (17)	0.017 (2)	−0.0066 (19)
C5	0.079 (2)	0.0471 (19)	0.099 (3)	−0.0049 (17)	0.014 (2)	−0.0124 (19)
C6	0.0514 (16)	0.0443 (16)	0.071 (2)	−0.0049 (13)	0.0061 (15)	−0.0082 (15)
C7	0.0439 (14)	0.0431 (15)	0.0444 (16)	0.0025 (12)	0.0026 (12)	−0.0018 (13)
C8	0.0398 (14)	0.0494 (16)	0.0431 (16)	−0.0029 (12)	−0.0011 (12)	−0.0026 (13)
C9	0.0357 (13)	0.0474 (15)	0.0413 (15)	0.0026 (11)	0.0015 (11)	0.0066 (12)
C10	0.0425 (16)	0.0648 (19)	0.0514 (18)	−0.0019 (13)	−0.0037 (13)	0.0026 (15)
C11	0.0344 (15)	0.075 (2)	0.069 (2)	0.0045 (15)	−0.0078 (14)	0.0192 (19)
C12	0.0478 (17)	0.064 (2)	0.098 (3)	0.0205 (16)	0.0038 (18)	0.019 (2)
C13	0.0557 (18)	0.0471 (17)	0.085 (3)	0.0122 (15)	−0.0055 (17)	0.0012 (17)
C14	0.0398 (14)	0.0406 (15)	0.0562 (18)	0.0013 (12)	−0.0031 (13)	0.0105 (13)
Cl3	0.0805 (7)	0.0978 (9)	0.1336 (11)	0.0076 (6)	−0.0101 (7)	−0.0009 (8)
Cl2	0.0855 (7)	0.1220 (10)	0.0938 (8)	0.0107 (7)	0.0185 (6)	−0.0140 (7)
Cl1	0.1125 (9)	0.0812 (7)	0.1130 (10)	−0.0309 (6)	0.0068 (8)	−0.0142 (7)
C30	0.068 (2)	0.059 (2)	0.071 (2)	−0.0078 (16)	0.0122 (17)	0.0078 (18)

### Geometric parameters (Å, º)

**Table:**

Zn—O	1.9472 (19)	C5—H5A	0.9300
Zn—O^i^	1.9472 (19)	C6—C7	1.393 (4)
Zn—N^i^	2.054 (2)	C6—H6A	0.9300
Zn—N	2.054 (2)	C8—C9	1.422 (4)
S—C2	1.761 (3)	C8—H8A	0.9300
S—C1	1.788 (3)	C9—C10	1.417 (4)
N—C8	1.297 (3)	C9—C14	1.420 (4)
N—C7	1.427 (3)	C10—C11	1.357 (5)
O—C14	1.309 (3)	C10—H10A	0.9300
C1—H1A	0.9600	C11—C12	1.380 (5)
C1—H1B	0.9600	C11—H11A	0.9300
C1—H1C	0.9600	C12—C13	1.382 (5)
C2—C3	1.396 (4)	C12—H12A	0.9300
C2—C7	1.396 (4)	C13—C14	1.402 (4)
C3—C4	1.376 (5)	C13—H13A	0.9300
C3—H3A	0.9300	Cl3—C30	1.748 (4)
C4—C5	1.385 (5)	Cl2—C30	1.751 (4)
C4—H4A	0.9300	Cl1—C30	1.749 (4)
C5—C6	1.380 (5)	C30—H30A	0.9800
			
O—Zn—O^i^	88.54 (11)	C7—C6—H6A	119.7
O—Zn—N^i^	146.08 (10)	C6—C7—C2	119.8 (3)
O^i^—Zn—N^i^	93.12 (9)	C6—C7—N	121.1 (3)
O—Zn—N	93.12 (9)	C2—C7—N	119.0 (2)
O^i^—Zn—N	146.08 (10)	N—C8—C9	128.0 (3)
N^i^—Zn—N	103.74 (12)	N—C8—H8A	116.0
C2—S—C1	102.40 (16)	C9—C8—H8A	116.0
C8—N—C7	117.7 (2)	C10—C9—C14	118.8 (3)
C8—N—Zn	120.54 (19)	C10—C9—C8	116.0 (3)
C7—N—Zn	121.25 (16)	C14—C9—C8	125.1 (2)
C14—O—Zn	125.24 (17)	C11—C10—C9	122.4 (3)
S—C1—H1A	109.5	C11—C10—H10A	118.8
S—C1—H1B	109.5	C9—C10—H10A	118.8
H1A—C1—H1B	109.5	C10—C11—C12	118.4 (3)
S—C1—H1C	109.5	C10—C11—H11A	120.8
H1A—C1—H1C	109.5	C12—C11—H11A	120.8
H1B—C1—H1C	109.5	C13—C12—C11	121.7 (3)
C3—C2—C7	118.9 (3)	C13—C12—H12A	119.2
C3—C2—S	123.2 (2)	C11—C12—H12A	119.2
C7—C2—S	117.9 (2)	C12—C13—C14	121.0 (3)
C4—C3—C2	120.8 (3)	C12—C13—H13A	119.5
C4—C3—H3A	119.6	C14—C13—H13A	119.5
C2—C3—H3A	119.6	O—C14—C13	119.2 (3)
C3—C4—C5	120.2 (3)	O—C14—C9	123.2 (3)
C3—C4—H4A	119.9	C13—C14—C9	117.6 (3)
C5—C4—H4A	119.9	Cl1—C30—Cl2	110.6 (2)
C6—C5—C4	119.8 (3)	Cl1—C30—Cl3	110.7 (2)
C6—C5—H5A	120.1	Cl2—C30—Cl3	110.5 (2)
C4—C5—H5A	120.1	Cl1—C30—H30A	108.3
C5—C6—C7	120.6 (3)	Cl2—C30—H30A	108.3
C5—C6—H6A	119.7	Cl3—C30—H30A	108.3

Symmetry code: (i) −*x*, *y*, −*z*+1/2.
